# X-CHAR: A Concept-based Explainable Complex Human Activity Recognition Model

**DOI:** 10.1145/3580804

**Published:** 2023-03-28

**Authors:** JEYA VIKRANTH JEYAKUMAR, ANKUR SARKER, LUIS ANTONIO GARCIA, MANI SRIVASTAVA

**Affiliations:** University of California Los Angeles, USA; University of California Los Angeles, USA; University of Southern California, Information Sciences Institute, USA; University of California Los Angeles, USA

**Keywords:** Activity recognition, Neural networks, Explainable AI, Interpretability

## Abstract

End-to-end deep learning models are increasingly applied to safety-critical human activity recognition (HAR) applications, e.g., healthcare monitoring and smart home control, to reduce developer burden and increase the performance and robustness of prediction models. However, integrating HAR models in safety-critical applications requires trust, and recent approaches have aimed to balance the performance of deep learning models with explainable decision-making for complex activity recognition. Prior works have exploited the compositionality of complex HAR (i.e., higher-level activities composed of lower-level activities) to form models with symbolic interfaces, such as concept-bottleneck architectures, that facilitate inherently interpretable models. However, feature engineering for symbolic concepts–as well as the relationship between the concepts–requires precise annotation of lower-level activities by domain experts, usually with fixed time windows, all of which induce a heavy and error-prone workload on the domain expert. In this paper, we introduce *X-CHAR* , an eXplainable Complex Human Activity Recognition model that doesn’t require precise annotation of low-level activities, offers explanations in the form of human-understandable, high-level concepts, while maintaining the robust performance of end-to-end deep learning models for time series data. *X-CHAR* learns to model complex activity recognition in the form of a sequence of concepts. For each classification, *X-CHAR* outputs a sequence of concepts and a counterfactual example as the explanation. We show that the sequence information of the concepts can be modeled using Connectionist Temporal Classification (CTC) loss without having accurate start and end times of low-level annotations in the training dataset–significantly reducing developer burden. We evaluate our model on several complex activity datasets and demonstrate that our model offers explanations without compromising the prediction accuracy in comparison to baseline models. Finally, we conducted a mechanical Turk study to show that the explanations provided by our model are more understandable than the explanations from existing methods for complex activity recognition.

## 1 INTRODUCTION

Ubiquitous sensors such as those found in smartphones and wearables enable emergent sensing applications across all facets of society. In particular, Human Activity Recognition (HAR) has gained significant importance over the past few years in a variety of applications such as health and fitness monitoring [37, 51], remote patient care [42], and smart homes [20]. Modern HAR applications opportunistically combine multiple sensing streams to boost performance and exceed the potential when using each sensor in isolation. Traditionally, researchers used to handcraft a compositional set of features from the sensory data and used classical Machine Learning techniques such as support vector machine (SVM) and linear models for HAR [14, 23, 24, 39, 62]. On the other hand, recent research has shown that extracting specific features from the raw sensor values is an unnecessary burden on the developer as deep neural networks (DNNs) can automatically learn intermediate representations for decision-making. Furthermore, several works have shown that DNNs achieve better performance in activity recognition tasks when compared to traditional methods [47, 60]. However, DNNs provide performance and ease of use at the expense of explainable decision-making.

DNNs, by design, are black-box in nature. The superhuman ability to identify patterns purposefully exceed human reasoning capabilities. However, understanding the model’s decision by the end-user is critical in several domains – particularly those involving high-stake decisions. Moreover, several governmental agencies are slowly proceeding to regulate AI to be more transparent. The first to move in this direction is that the countries of the European Union have set several guidelines that state that any AI-based system should be completely explainable [2, 3].

In an attempt to provide insight into a DNN model’s inference after training, previous works have introduced various techniques called post-hoc explanation methods. For a given test input and a trained DNN model, these methods generate useful approximations of the model’s inner working and decision logic by producing understandable representations in the form of feature importance scores, rule sets, heatmaps, or natural language. But, unlike images or text, sensory data such as motion sensors are multivariate time series and are inherently non-readable by an average end-user. Moreover, most of the post-hoc explanations are not trustworthy [49], and they provide incomplete explanations that are not faithful to what the original model computes. For example, saliency-based post-hoc methods [52, 53] give an incomplete view of the decision-making process as they only highlight where the model is looking at while making a decision without explaining the full reasoning. Therefore, highlighting specific segments on the input sample will not help understand the model’s decision. Finally, even if one could effectively visualize activations over opaque sensor data, the problem is further exacerbated when considering multiple heterogeneous streams of unreadable sensor data. Thus, we strive to compose explanations for complex activity recognition models analogous to how domain experts would engineer robust and interpretable features across sensor streams to explain to a layperson without domain expertise.

In this paper, we exploit the compositionality of complex events to develop inherently interpretable deep learning models that can learn human-understandable representations in the latent space. In particular, we take inspiration from concept-bottleneck models [31] that provide explanations in the form of human-understandable, high-level “concepts." For instance, instead of end-to-end bird species classification, the model first learns to identify intermediate concepts such as beak color and wing length. However, these models primarily focused on image classification models. For complex activity recognition tasks, several challenges need to be addressed to generate meaningful explanations in the form of concepts.

### 1.1 Challenges for Concept-based Explainable Human Activity Recognition

**Fig. 1.**
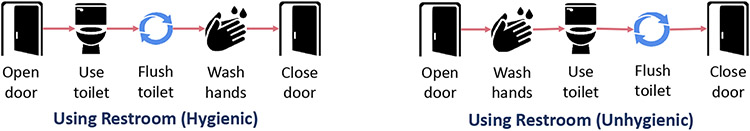
Examples of complex activities.

We formalize a complex activity (e.g., hygienic restroom usage) as a composition of lower-level concepts (e.g., washing hands). Thus, the first challenge in explaining complex activity detection requires identifying the correct *sequence of concepts*. The existing concept bottleneck model architecture [31] currently provides only the presence or absence of concepts as an explanation for image classification. However, complex activity models observe time series data and the sequence and frequency in which concepts are present or absent matter to make the final complex activity inference. For example, as shown in Figure 1, for a person to use the restroom hygienically, they must perform the following sequence of concepts: enter restroom-> use toilet -> flush toilet -> wash hands -> exit restroom. If the person does not wash their hands after using the restroom, it should be flagged as an unhygienic use of the restroom. Second, the concept extraction process from the raw signals is not trivial. The concepts for a complex activity can vary in length. They can range from less than a second to a few seconds long. Some parts of the input time series also carry random signal values which do not have any semantic meaning for a particular task and hence should not be detected for that particular task. Third, it is usually difficult to have an accurate alignment (i.e., correspondence of the elements) of the input sequence and the concepts. That is, while collecting data for complex activities, it is easy to annotate the sequence of concepts that occurred rather than accurately annotating the start and end times of every concept. In fact, several existing works [25, 32] have shown that the start and end-times in public HAR datasets are not accurate as there are always some time jitters in between annotating and performing the activity.

### 1.2 Contributions

**Fig. 2.**
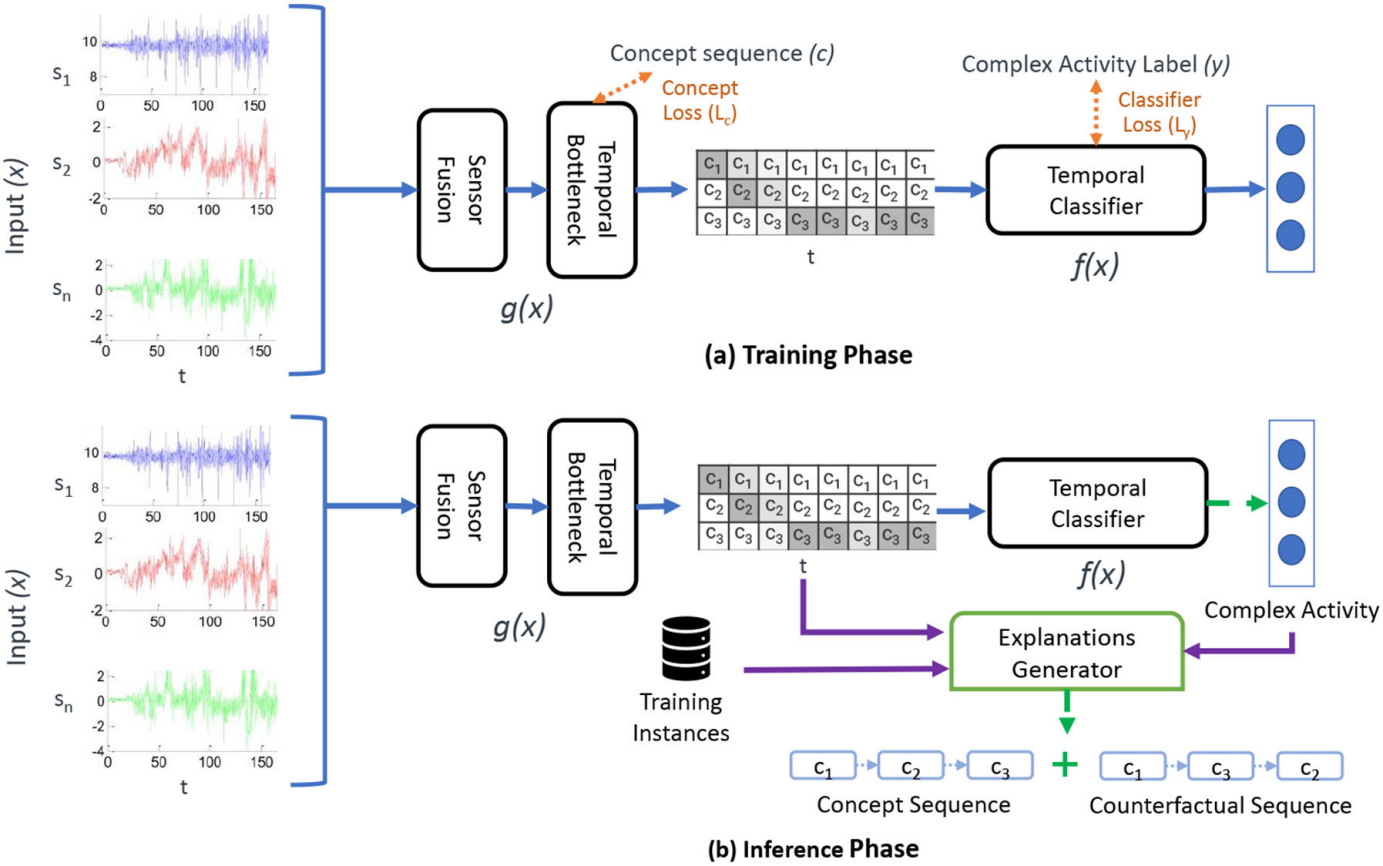
An overview of the proposed end-to-end *X-CHAR* model during the training and inference phases.

To this end, we propose X-CHAR (eXplainable Complex Human Activity Recognition), an end-to-end inherently interpretable deep learning model that not only infers the complex activity given an input sensor stream but also provides an explanation based a sequence of concepts that is responsible for the particular classification. In particular, the model first identifies simple activities relevant to the particular task as concepts whose sequential ordering will compose a complex activity. Figure 2 shows the different components of the proposed end-to-end interpretable deep learning model. X-CHAR consists of a sensor fusion model that combines information from multiple sensors, an intermediate *Temporal Bottleneck* layer to identify the concepts and a final classifier to predict the complex activity. Since the input sensor data and the concepts are unaligned, not annotated with timestamps and can be of variable length, we use the *Connectionist Temporal Classification* (CTC) loss to train the bottleneck layer and solve the challenges mentioned. Finally, X-CHAR recognizes the complex activity and provides an explanation by generating counterfactual explanations based on the decoded concepts and the given complex activity classification. The counterfactual explanation provides a faithful explanation of the model’s decision using human-understandable concepts while highlighting the importance of the temporal ordering of the concepts.

To demonstrate the efficacy of our approach, we use three complex activity datasets (i.e., Nurse Activity, Opportunity, and Complex Restaurant Activities datasets) where the classification tasks require an inference explanation. We first compare our model against the existing standard end-to-end deep-learning methods for activity classification and show that our architecture provides additional benefits of an inherently interpretable model without impacting performance (i.e., accuracy). We then conduct a user study to show that humans perceive the explanations (in the form of concepts) as good explanations for the classification.

To summarize, the major contributions of this paper are as follows:

- We propose X-CHAR, an end-to-end inherently interpretable DNN model architecture for complex activity recognition.
- We show that the sequence information of the concepts can be modeled using CTC Loss without having accurate start and end times in the training dataset.
- We show that having a temporal bottleneck layer does not decrease the task accuracy compared to baseline models and has the additional benefit of being interpretable.
- We provide faithful explanations of complex activity model predictions based on human-understandable concepts while highlighting the importance of the sequential ordering of the concepts.
- We evaluate our approach on multiple time series datasets. We conducted extensive experiments to show that adding this bottleneck layer provides explainability with no loss in performance. We also conducted a human study to show that these explanations are preferred over other baseline explanation methods.

The rest of this paper is organized as follows. We discuss related literature in Section 2. In Section 3, we define the concepts, complex activities and the problem statement. In Section 4, we first provide an overview of X-CHAR framework and explain its building blocks in detail. Then in Section 5 we discuss the datasets and baselines that will be used in evaluating X-CHAR . We evaluate and compare X-CHAR ’s performance and explainability with other baselines in Section 6. In Section 7, we discuss the results, limitations of X-CHAR and draw some future research directions. Finally, we provide a summary of the benefits and applications of our work in Section 8.

## 2 RELATED WORKS

### 2.1 Human Activity Recognition

Human Activity Recognition (HAR) using sensory data has gained significant importance over the past few years. It is being used in a variety of applications like health and fitness monitoring [37, 51], remote patient care [42], smart homes [20] etc. Research has shown that data from motion sensors, microphones, and ambient sensors can be used effectively to classify various day-to-day activities of humans [4, 47, 56] using temporal convolutional neural network and recurrent neural network architectures. Several existing works [27, 28, 60] show that the combinations of convolutional and Long-Short Term Memory (LSTM) layers achieve better performance in complex activity recognition tasks when compared to the traditional statistical-based methods [14, 24, 39, 62].

### 2.2 Complex Human Activity Recognition

These methods mainly fall under two broad categories. Given the data from different sensors, i) They predict only the complex activities or ii) They predict simple activities and then hierarchically infer complex activities.

#### 2.2.1 Complex Activity Recognition

Cho *et al*. [13] showed the use of convolutional neural networks to fuse different modalities and to detect complex activities. Qin *et al*. [46] used a residual neural network architecture to extract the features from sensory data. Similarly, Chen *et al*. [12] utilized Long-Short Term Memory (LSTM) models to capture the temporal information from the sensors to predict complex activities. Francisco et al. [41] used a hybrid model consisting of both convolutional layers and recurrent layers that can capture the Spatio-temporal relationships in the data to infer the activities.

#### 2.2.2 Hierarchical Complex Activity Recognition

Peng *et al*. [44] proposed a hybrid activity recognition model, AROMA, utilizing a CNN along with LSTM models. The authors used CNN to extract task-specific features from sensor data and then utilized an LSTM network to learn the temporal context of activity data. Xia *et al*. [57] proposed complex activity recognition models by utilizing sensor data *motifs*, which discretize sensor signals into symbols that can be fed into sequencing models. However, these works on complex activity recognition require accurate labeling of sensory time series data, which is tedious. Also, they do not explain or interpret the recognition outcomes. Additionally, the motifs-based models are not designed with interpretability in mind, as they represent low-level pre-processing features of sensor signals independent of the target domain.

### 2.3 Explainable Deep Learning

Since neural networks are black boxes, many different approaches have been developed in recent years to explain the working of the models. These methods broadly fall under two categories: i) Post-hoc methods ii) Inherently interpretable methods.

#### 2.3.1 Post-hoc Explanations

These methods generate explanations in the form of simple linear models, shallow decision trees, or even visualizations that assist stakeholders in comprehending how the model works. They extract correlations between feature values and predictions and approximate the behavior of a black box model. LIME [48] provides explanations by approximating the local decision boundary by a simple, sparse linear model to simulate the prediction. SHAP [36] uses a game-theoretic approach to identify the importance of different features for a prediction to provide necessary explanations. There are several works [40, 52-54, 63] that provide explanations in the form of saliency maps over the input sample, highlighting the important input regions. The saliency map is obtained using the gradient information of output with respect to the input features. GradCAM [52] is a method designed particularly for convolutional neural networks. It produces a coarse localization map by utilizing the activation maps of different convolutional filters and highlighting the image’s important regions. Explanation by prototypes [29, 30, 34] methods project explanations across the underlying training data. They provide the nearest matching data samples from the training dataset as representative examples to explain the model’s prediction, and they mostly use different distance metrics (i.e., Cosine, Euclidean, and Manhattan) to get the similarity scores. Another interesting post-hoc explanation method is the Counterfactual explanation [55]. Counterfactual explanation describes a minimal change to the input that would result in the opposite prediction. While there have been multiple ways to generate counterfactual explanations proposed in the literature, they are mainly designed for images [9], text [59], and tabular data [43] as they are easy to modify and are human-understandable. However, for time series data like inertial sensors, just perturbing the input would not be helpful as the data is non-readable and hence the counterfactual would be non-interpretable. Ates et al. [6] and Carvalho et al. [8] did propose methods for generating counterfactuals for time series but did not do a human study to evaluate the comprehensibility of the explanations. In general, The explanations from the post-hoc methods are incomplete and require human supervision to generate the final explanations with proper domain expertise. Also, research has shown that most post-hoc explanations are not faithful to the model or the predictions.

#### 2.3.2 Inherently Interpretable Models

Traditional machine learning models like Decision trees and Linear models fall under this category. In recent years, researchers have been aiming to design interpretable neural networks by imposing interpretability restrictions during the network training process. For example, Zhang et al. [64] proposed an interpretable CNN with a novel loss term that encourages each high-layer convolutional filter to represent a specific part of the object. Another common approach is integrating attention into neural networks [21, 65]. These models aim to expose the parts of an input the model focuses on for the final decision-making tasks. Concept bottleneck models [18, 31, 61] (CBM) are recently being used for image classification, retinal disease prediction, and visual question-answering tasks. These are end-to-end models that first predict the concepts in the latent spaces, then use only those predicted concepts to make a final prediction. The main limitation of the existing CBM is that it only provides the presence or absence of a concept without any proper sequencing of these concepts, which is vital for activity recognition in critical domains (e.g., hospital patient caring).

### 2.4 Explanations for Activity Recognition

Unlike images or text, the data for HAR is a multivariate time series obtained from multiple sensors, including inertial motion sensors that are not human-readable. So, the methods to explain Activity recognition broadly fall under two categories: 1) Methods that explain the activity recognition on the raw input space and 2) Methods that explain activity recognition in a human-understandable concept space. Most of the existing works for explainable HAR fall in the first category. Das *et al*. [16] compared the different post-hoc explanation methods for simple activity recognition where certain regions in the input sensory data are highlighted. Schlegel et al. [50] showed how to apply saliency-based post-hoc methods to time series data, but the methods were not evaluated on their understandability to end-users. However, more recent works are exploring methods that fall in the second category. Arrotta *et al*. [5] proposed DeXAR, which converts sensory data into human-readable images and then applies post-hoc explanation methods. However, since they use post-hoc methods to explain the CNN classifier, the explanations obtained are heatmaps, which have to be processed in an application-specific way to generate human-understandable text explanations. Also, identifying the suitable threshold for selecting the important segments for each test input is challenging. Neuroplex [58] combines the neural network with reasoning layers for detecting complex events from simple activities. However, their method requires human knowledge to be provided during training and cannot handle data from multiple sensors, and is limited to a single sensor. Also, it assumes that we have accurate time information for all the simple activities, which is not practical in a real-world scenario.

Therefore, unlike prior works, our *X-CHAR* model is an end-to-end, inherently interpretable DNN model that captures the concept sequence for complex human activity recognition. It also provides counterfactual explanations in the human-understandable concept space, thereby making the explanations easy to comprehend. While training, our approach does not require accurate time information for simple activities or concepts, unlike the other methods. *X-CHAR* model learns the alignment of the concepts from the training data automatically.

## 3 BACKGROUND AND PROBLEM STATEMENT

In this section, we first define the concepts and their relationship to complex human activities. Then, we present the problem statement of our work.

### 3.1 Definitions

#### 3.1.1 Simple Activity

A simple activity is defined as a unit-level activity that can be captured by the given sensors within a short time window and cannot be broken down further, given application semantics. For example, the body-worn accelerometer sensor can capture ‘standing,’ ‘sitting,’ or ‘washing hands’ activities, which can not be further broken down. The level of granularity depends on the sampling frequency of the sensor. For instance, activities like ‘picking up an item’ require a high sampling frequency. In contrast, a low sampling frequency can capture activities like ‘sitting.’ Also, simple activities such as ‘standing,’ ‘sitting,’ ‘washing hands,’ and ‘oral care’ can occur sequentially or concurrently. The existing deep learning algorithms have shown remarkable performance in detecting simple activities.

#### 3.1.2 Concepts

In explainable AI literature, the definition of concepts with regard to images is; a group of pixels that represent a higher-level and human-understandable feature that is relevant to a particular application/task. For instance, in the bird species classification task, the wing and beak colors are considered the concepts, whereas the sky color or background is completely ignored for that application. In this paper, we define the concepts of a complex activity recognition task as follows. A concept is a section/portion of the input sequence that corresponds to a human-understandable simple activity and is relevant to the particular task. For example, in the nurse activity detection task, we consider blood collection and measuring vital signals as the concepts, whereas we completely ignore sitting or standing for that detection task. Also, a group of concepts has temporal dependencies: sequence/order of the concepts and the frequency of each concept in the sequence matter. Therefore, we can say that all concepts are simple activities, but not all simple activities are concepts. For instance, we care about the sequence of measuring vital signals, blood collections, and drips for safe drip procedure detection; however, we ignore any sitting or standing activity for the particular task.

#### 3.1.3 Complex Activity

In this paper, a complex event is strictly defined as a pattern or sequence of ≥ 2 instances of concepts with temporal dependencies. Under this definition, a complex event must be composed of multiple simple events that may evolve over long periods of time in different orders and frequencies. For example, as shown in Figure 1, the "Hygienic use of Restroom" activity is considered a complex activity that consists of five concepts: "opening door", "using toilet", "flushing", "washing hands", and "closing door". If the person does not wash their hands after using the restroom, then the complex activity is ’Unhygienic use of Restroom’. This shows that the sequence of the concepts matters for a particular complex activity. Another example is a sanitary protocol violation event in a hospital scenario: a nurse could violate the sanitary protocol if they process one patient and then processes another patient without proper sanitation.

### 3.2 Problem Formulation

We consider the problem of predicting a complex activity label y∈Y, where Y is the set of complex activity labels of size L, from a multivariate time-series data input $x\in R^{S\times T}$, where S is the number sensors and T is the time sample window. We observe training dataset, $\Phi ={\{(x^{\left(i\right)},y^{\left(i\right)},c^{\left(i\right)})\}\;}_{i=1}^{N}$ , where each $c^{\left(i\right)}$ is a sequence of concepts such that $c^{\left(i\right)}=[c_{i,1}\to c_{i,2}\to \ldots \to c_{i,k_{i}}]$ and $k_{i}$ is a scalar, variable number of concepts for each input, and where $k_{i}<<T$ and $c_{i,k}\in C$, where C is a set of unique concepts in the dataset of size M. Similar to prior bottleneck models, our goal is to learn a function f(g(x)), where g(x) maps an input x into a concept space. In our case, $g\;:R^{S\times T}\to R^{M\times T}$, and $f\;:R^{M\times T}\to R^{1\times L}$. Intuitively, at every sample window of size T, g(x) maps the sensor input to a matrix of concept scores of size M×T, which is then mapped by f to an output complex activity label. However, the output of g(x) only provides a matrix of concept scores, where as our goal is to output the complex activity label and the sequence of concepts that compose the complex activity decision. Thus, we also use an explanation generator function e(g(x,Φ)) that decodes the output of g(x) to the concept sequence and obtains a counterfactual instance, which is then presented as the explanation for the complex activity decision. Figure 2 shows the overview of our proposed solution, *X-CHAR* , for this problem statement.

## 4 SYSTEM DESIGN

In this section, we provide an overview of X-CHAR (Section 4.1) architecture. We then present the detailed descriptions of the different building blocks of the proposed system with appropriate figures, descriptions, and algorithms (as in Sections 4.1.1, 4.1.2, 4.1.3).

### 4.1 X-CHAR Model Overview

**Fig. 3.**
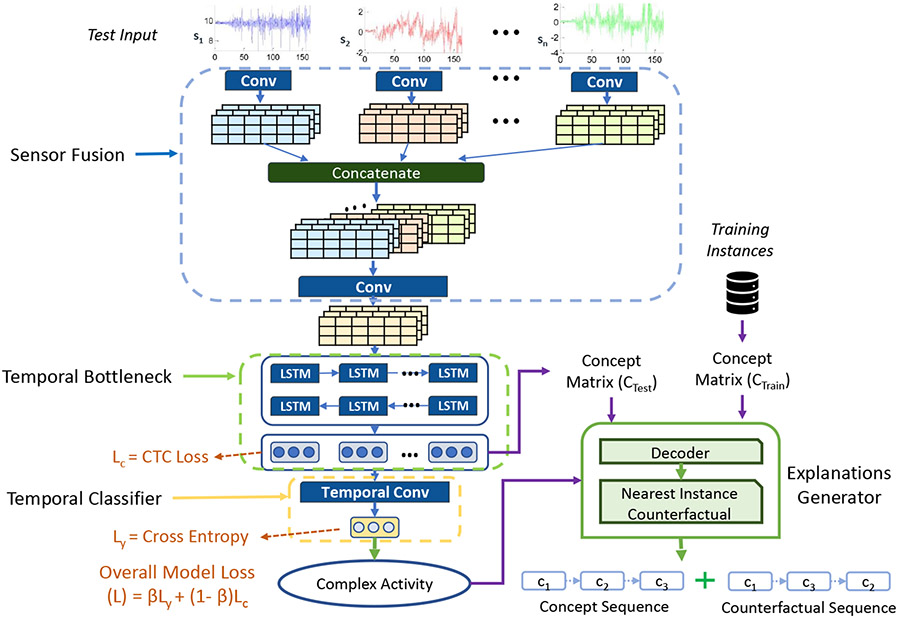
The overall *X-CHAR* model design showing the various layers in each module.

This work introduces *X-CHAR* , an interpretable DNN architecture for the concept-based complex activity classification that provides both the complex activity classification label and its corresponding explanation in the form of a concept sequence for a given input sensory data. Figure 3 depicts the overall model design that shows how both the classification label and the corresponding explanation are generated, *X-CHAR* . There are three integral parts: Sensor fusion module, temporal bottleneck module, and classification module in *X-CHAR . First*, the Sensor fusion module extracts features from different sensors and maps them to shared latent space. *Second*, the temporal bottleneck module extracts the temporal relationship between the obtained features and predicts the concept associated with each timestep. *Third*, the classification module predicts the final complex activity label from the concepts. Each of these modules is described below in detail.

#### 4.1.1 Sensor Fusion Module

This module consists of two steps: the first step extracts intra-sensor features, and the second step extracts the inter-sensor features. In the first step, each sensor stream is considered separately and passed through a series of one-dimensional convolutional layers (1-D Conv). The 1-D convolutional layer is used to extract local features from 1D patches in every sensor sequence to identify local patterns within the convolution window. Since the same transformation is applied on every patch identified by the window, a pattern learned at one position can also be recognized at another position, making 1D convolution layers translation invariant. The input to the first stage is input $X_{i}$ which has the shape of [S×t]. Here S is the number of sensory channels in the input stream. The output of the first stage is a collection of feature maps corresponding to each filter for every sequence, which is of the shape [$S\times t\times f_{1}$]. Here $f_{1}$ is the number of 1-D kernels. Next, the sensor fusion model concatenates the feature maps to the shape of [$t\times (S\times f_{1})$] and feeds it to the second set of convolutional layers. This layer captures the inter-sensory features between the different sensor streams. Therefore, the output of the second convolutional layer is of the shape [$t\times f_{2}$]. Here $f_{2}$ is the number of convolution kernels in the second convolutional layer.

#### 4.1.2 Temporal Concept Bottleneck Module

Let us recall the key challenges discussed in Section 1.1; the model has to capture the temporal relationship of the concepts, the length of the concepts is not fixed, and the exact accurate alignment (i.e., correspondence of the elements) of the input sequence and the concepts is also unknown. To solve these challenges, we design the *Temporal Concept Bottleneck* module and train it with Connectionist Temporal Classification [22] (CTC) loss to facilitate the proper alignment between the input sequence and output concepts.

This module is designed to identify the sequence of concepts $C_{i}$ from the given input $X_{i}$. This module has three main components: a set of bi-directional LSTM layers, a time-distributed dense layer, and a softmax activation layer. First, this module uses a set of bi-directional LSTM layers to capture the temporal information from the preceding sensor fusion module. Next, the time-distributed dense layer maps the temporal features from the proceeding module to different concepts to aid the softmax activation layer. The total number of neurons M in the dense layer equals the number of unique concepts m present in the dataset. Finally, the softmax layer outputs the probability distribution over the possible concepts at each timestep t.

##### Concept Loss:

We use ***Connectionist Temporal Classification*** [22] or CTC Loss to train the temporal bottleneck module. CTC loss is designed for tasks where we need to predict the alignment (correspondence of the elements) between the input sequence and target sequence, but that alignment information is not present while training the model. In a complex activity recognition task, since we do not have an accurate alignment of input sample X and the concept sequence C in the training dataset, we use CTC loss to model the concepts. It calculates a loss between a continuous (unsegmented) time series and a target concept sequence by summing over the probability of possible alignments of input to concepts, producing a differentiable loss value with respect to each input node. Thus, the CTC alignments provide a natural way to go from probabilities at each time step to the probability of an output sequence. We get the probability for any $C_{i}$ given an $X_{i}$ as shown in Equation (1).

(1) $$p(C_{i}|X_{i})=\sum_{a\in A_{X_{i},C_{i}}}\prod_{t=1}^{T}p_{t}(a_{t}|X_{i})$$

where ($X_{i},C_{i}$) is the ’i’th pair of input and concept sequence, $A_{X_{i},C_{i}}$ is set of the alignment between ($X_{i},C_{i}$) and $p_{t}(a_{t}|X_{i})$ is the probability of alignment of $a_{t}$ given the input instance $X_{i}$. We convert the probability of the concept sequence into a loss function by taking the negative logarithm as shown in Equation (2)

Also, as discussed in the challenges in Section 1.1, it does not make sense to force every input time-step to align to some concept as there might be some random motion between activities. CTC Loss addresses this issue by introducing a new token to the set of allowed concepts. This new token is called the blank ’ϵ’ token. The ϵ token does not correspond to anything and is simply removed from the inference phase as discussed in Section 4.3.


(2) $$L_{C}=\sum_{C_{i},X_{i}}-\;\log\;p(C_{i}|X_{i})$$

#### 4.1.3 Classifier Module

This module predicts the final complex activity given the concepts from the temporal bottleneck module. This module uses a Temporal Convolutional (TCN) layer followed by a dense layer with softmax activation. The TCN performs dilated causal convolutions and captures the relationship between the concepts from the temporal bottleneck module, and the dense layer predicts the complex activity. The dense layer has ’L’ neurons corresponding to the number of output classes. And since this is a classification problem, we use softmax (σ) as the activation of the final dense layer, which is given in Equation (4). The softmax function imparts probabilities to the logits ’s’ when we have multiple classes, and we get the probability distribution of output classes. We consider the most probable occurrence with respect to other outputs as the predicted class.

##### Classification Loss:

Since it is a multi-class classification problem, we use categorical cross-entropy as our complex activity loss function. The loss is calculated as per Equation (3)

(3) $$L_{Y}=-\sum_{j=1}^{n}y_{j}\;\log\;\sigma (s_{j})$$

(4) $$\text{where},\;\;\sigma (s_{i})=\frac{e^{s_{i}}}{\sum_{j=1}^{n}e^{s_{j}}}$$

### 4.2 Training Phase

The entire X-CHAR classification model is trained in an end-to-end manner. Therefore, the overall loss of the model (L) is a weighted sum of concepts loss (Lc) and classification loss (Ly) as written in Equations (5) as

(5) $$L=\beta L_{Y}+(1-\beta )L_{C}$$

where β is a hyper-parameter. Therefore, the model is trained by jointly reducing both the loss functions. In our experiments, we found that β=0.5 that gives equal importance to both concept and classification loss achieved the best performance.

### 4.3 Generating Explanations

#### 4.3.1 Complex Activity Classification

After we have trained the *X-CHAR* model, we use it to predict the complex activity and find the likely concept sequence to explain a given input sensory data. The predicted activity is obtained by taking the argmax of the probabilities from the final dense layer of the classifier module.

#### 4.3.2 Generating Concept Sequence:

**Fig. 4.**
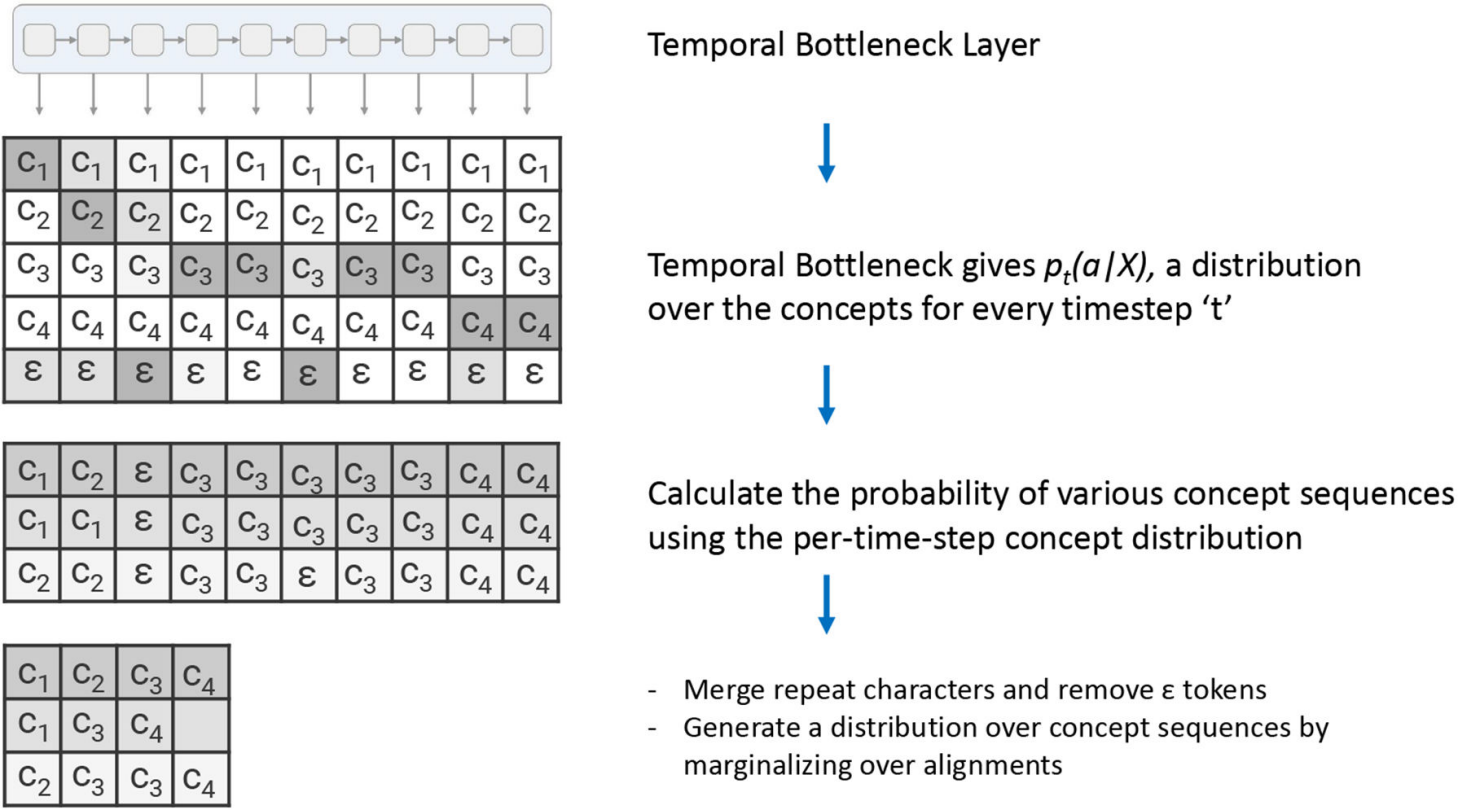
The concepts decoder: Converts the probability distribution of the concepts at every timestep to the most probable concept sequence for a given input.

The corresponding concepts sequence is obtained by decoding the output of the temporal bottleneck module with the beam search algorithm [35]. The concept decoder steps are shown in Figure 4. The temporal bottleneck module gives the concept matrix, which is a probability distribution of concepts at every timestep, t. Next, we find the probability of various concept alignments from the per-time-step probabilities. But this can be very expensive to compute as there will be a massive number of alignments. Hence, we use the beam search algorithm to calculate the sequence probabilities, significantly reducing the time complexity. Then, the decoder removes duplicate concepts (occurring one after another) and then removes all blanks from the path resulting in concept sequences for each alignment. Since it is possible for multiple alignments to have the same resulting concept sequence, the decoder generates the probability of different concept sequences by marginalizing over alignments. Finally, the concept sequence with the highest probability is obtained.

#### 4.3.3 Generating Counterfactual Explanation:

We generate counterfactual explanations using a method similar to the Nearest Instance Counterfactual Explanations method [7] but modified for temporal sequences. We propose using ***Damerau–Levenshtein distance*** [15] as the proximity metric to measure the distance between the concept sequences. The Damerau–Levenshtein distance between two concept sequences is the minimum number of operations (consisting of insertions, deletions or substitutions of a single concept, or transposition of two adjacent concepts) required to change one concept sequence into the other. This method provides explanations by using instances from the underlying training data in contrast to the methods that alter the inputs to generate counterfactuals.

The main reasons for adopting this method of counterfactuals are: (i) Plausibility: The input data can be modified if infinite ways, but not all modifications are plausible in real-life scenarios, especially with regard to activity sequences. Hence, making use of training instances ensures that the generated counterfactuals are plausible (ii) Proximity metric: Existing methods use Euclidean or Manhattan distance to measure proximity, whereas, for sequences, the distance is the number of operations required to change from one sequence to another. Hence, we use the proposed Damerau–Levenshtein distance .

##### Method:

The counterfactual explanations are generated by the following steps:

- Step 1: Initialization - Given Training dataset Φ, pass it through the *X-CHAR* ’s complex activity recognition model. Then obtain and store the concept sequence for all the samples in the training dataset, $C_{\Phi }$.
- Step 2: Given a test input $x_{test}$, obtain the concept sequence $c_{xtest}$.
- Step 3: Find the nearest neighbor $c_{ex}$ in $C_{\Phi }$ which has the minimum Damerau–Levenshtein distance from $c_{xtest}$ and is classified as a different class. $c_{ex}$ is the generated counterfactual explanation.

Step 1 has to be done only once as $C_{\Phi }$ is constant and will be stored. So for the new test samples, we can skip step 1. Explanation from *X-CHAR* for an input instance is shown in Figure 8.

## 5 IMPLEMENTATIONS

This section introduces the three complex activity datasets to test our *X-CHAR* model. Then, we discuss the comparison of baseline models and baseline explanation methods. Finally, we also mention the metrics used in comparing *X-CHAR* with the baseline methods.

### 5.1 Datasets

In this paper, we used three different complex activity datasets as described in the following paragraphs:

#### 5.1.1 Complex Nursing Activity Dataset

The complex nursing event dataset is based on a public dataset from Nursing Activity Recognition. Challenge [33]. The dataset contains nurse activity information from an accelerometer sensor, and it includes six different simple activities performed by eight individuals (i.e., nurses). The simple activities are: (i) Vital signs measurements, (ii) Blood collection, (iii) Blood glucose measurement, (iv) Indwelling drip retention and connection, (v) Oral care, and (vi) Diaper exchange and cleaning of the area. The duration of each data segment ranges from 30 to 60 seconds. These simple activities are considered as the concepts in our experiments. The sampling frequency of the accelerometer is 4Hz. The dataset is split into two parts: the first part contains data from six nurses, and the second part contains data from the remaining two nurses. We then generate five complex nursing activities data by randomly selecting segments corresponding to the different concepts and concatenating them together in a predefined order for each complex activity, as mentioned in Table 1. We generated a training dataset of 3000 complex activity samples (i.e., 600 for each complex activity) from the first part and a validation dataset of 1000 complex activity samples (i.e., 200 for each complex activity) from the second part. The evaluations reported in Section 6 are based on the comparison methods’ performance on the validation dataset.

#### 5.1.2 Opportunity Dataset

This dataset was compiled from five separate subjects, with data taken at four different periods for each. Body motion sensors, object sensors, and environmental sensors are among the sensors in the dataset. We consider the inertial body-worn sensors worn in five different positions on an individual: left lower arm, left upper arm, right lower arm, right upper arm, and back of the torso because we focus on activity detection. These inertial units record the accelerometer, gyroscope, and magnetometer data. All activities are divided into two categories: high-level activities (early morning routine, preparing a sandwich, making tea, and cleaning) and low-level activities (which include 17 different micro-tasks such as opening and closing doors, shelves, and so on). We considered data from four users in training and the fifth user in testing. A brief description of the dataset is shown in Table 2

#### 5.1.3 CRAA: Complex Restaurant Activities from Audio Dataset

This complex activity dataset is generated by using a subset of the audio samples from ESC-50 [45] and Kitchen-20 [38] audio datasets. The ESC-50 dataset is a labeled collection of environmental audio recordings that consists of 5-second-long recordings organized into 50 semantical classes. The kitchen20 dataset contains 5 to 10 seconds audio recordings from kitchen activities for 20 different classes. From these two datasets, we considered the audio clips from 11 different simple activities as the concepts in the paper. We then constructed an audio-based complex human activity recognition dataset by concatenating the concepts in different sequences to obtain the various complex events. In particular, we generated the following five complex activities performed by a person working in a restaurant: making a juice, making a puree/sauce, having a drink, and hygienic and in-hygienic uses of the restroom. In total, we synthesized a training dataset of 1000 complex activity audio samples (i.e., 200 for each complex activity) and a test dataset of 250 complex activity audio samples (i.e., 50 for each complex activity). Table 3 gives a description of the complex activities and their corresponding concept sequences in the CRAA dataset.

### 5.2 *X-CHAR* Model Architecture

Table 4 shows the model architecture of *X-CHAR* and the hyper-parameters, including the number of filters, neurons, and LSTM units in each layer. The best hyper-parameters were chosen through grid-search. We used the Adam optimizer to train the model.

### 5.3 Baseline Complex Activity Classification Models

We compared the performance of X-CHAR to four existing state-of-the-art complex activity prediction DNN models as follows:

- **ConvLSTM + TCN.** This is the non-end-to-end, single task version of the X-CHAR model. The ConvLSTM model is trained to predict the concepts. The TCN model is trained to predict the complex activity from the concepts. These models are trained independently and then operate in sequence during inference.
- **DEBONAIR** [11]. This is an end-to-end model for multimodal complex activity recognition. It uses convolutional layers to extract features from different modalities and LSTM layers to predict complex activities. This model only predicts the final complex activities and does not predict the concepts. Hence, it does not have a notion of interpretability.
- **AROMA** [44]. This is a multitask learning model that predicts both simple and complex activities. The input data is split into predefined smaller segments of equal size. We used the window size of 20 seconds as described in their work. Then each segment is predicted as a simple activity, and then a classifier predicts the complex activities from simple activities. This model requires the data to be labeled for every segment and does not consider that there will be segments that cannot be labeled or simple activities can be of different durations. Therefore, it does not predict simple activities correctly.
- **Concept-Bottleneck Models (CBM)** [31]. The CBM is a CNN-based model that predicts an intermediate set of human-specified concepts first, and then uses these concepts to predict the final label. This model is mainly designed for image classification tasks and only indicates the presence or absence of concepts and does not capture the temporal relations between them. For complex activity recognition, the sequence of simple activities matters.

### 5.4 Baseline Explanation Methods

The explanation methods for sensor-based Complex Human Activity Recognition can be categorized into two types: (1) Explanations projected on raw input space and (2) Explanations based on concepts.

#### 5.4.1 Explanations over Raw Input space

In this category, we considered the existing Gradient-weighted Class Activation Mapping (GradCAM) method and explanation by examples as our baselines as they have shown to be successfully applied to time-series classification applications with good understandability.

- **GradCAM** [52]. GradCAM is a saliency-based post-hoc explanation method. It uses the gradients of the predicted class propagating into the final convolutional layer to produce a coarse localization map in the form of a heatmap. This heatmap highlights the important regions in the input for predicting that class. But since sensory data is inherently non-readable for humans, just highlighting the important segments is not sufficient as an explanation.
- **ExMatchina** [29]. ExMatchina is an Explanation by Examples method that selects a particular set of ’k’ examples from the training dataset to explain the behavior of machine learning models. The ’k’ nearest examples were chosen from the training dataset by comparing feature activations at the last convolutional layer. These examples have the highest cosine similarity of their activation maps with the given text input. This method depends on the human reasoning ability to identify the commonalities between the examples and the text input. But since the sensory data is non-readable in nature, it is difficult to determine the similarities.

#### 5.4.2 Explanations over Concept space

In this second category, we considered explanations from CBM, AROMA, and DeXAR as baselines.

- **Concept-Bottleneck model** [31]. As discussed in 5.3, CBM is an interpretable DNN model that provides explanations in the form of high-level human-understandable concepts that accompany a prediction. They only indicate the presence and absence of concepts and do not provide the sequence or frequency information of the concepts.
- **DeXAR** [5]. DeXAR is an explanation method that transforms a sequence of simple activities into semantic images and obtains a heatmap of the simple activities to predict a complex activity. Then, it generates a natural language explanation based on the information from the heatmap.
- **AROMA** [44]. As discussed in 5.3, Aroma predicts a simple activity for every time step, which in turn predicts the complex activity. We provide the simple activities predicted by AROMA to explain the complex activity.

### 5.5 Metrics

#### 5.5.1 Normalized Confusion Matrix:

A confusion matrix is a tabular way of visualizing the performance of the prediction model. Each entry in a confusion matrix denotes the number of predictions made by the model where it classified the classes correctly or incorrectly. The “normalized” term means that each of these groupings is represented as having 1.00 samples. Thus, the sum of each row in a balanced and normalized confusion matrix is 1.00 because each row sum represents 100% of the elements in a class.

#### 5.5.2 Task Mean F1-score:

In our applications, the errors caused by false positives and false negatives are equally undesirable. Also, the distribution of classes in our datasets is not uniform. Therefore, we use the mean F1-score as our metric to compare the performance of the models. F1-score is the harmonic mean of precision and recall. The F1-scores for each class can be calculated using Equation 6.


(6) $$F1-score=2*\frac{precision*recall}{precision+recall}$$

To obtain the mean F1-score, we take the unweighted mean of the Fl-scores of all the classes.

#### 5.5.3 Concept Levenshtein distance (Edit Distance):

The Levenshtein distance or Edit distance is a metric for measuring the difference between two sequences. It counts the minimum number of operations (i.e., insertions, deletions, or substitutions) required to transform one sequence into the other. Since we predict the sequence of concepts, we use this metric to evaluate the concept prediction performance.

#### 5.5.4 Concept Accuracy:

Since not all the baseline models predict the sequence of concepts, we also use concept accuracy to compare the performance. Accuracy is the number of correct predictions made as a ratio of all predictions made.

## 6 RESULTS

### 6.1 *X-CHAR* Model Performance

**Fig. 5.**
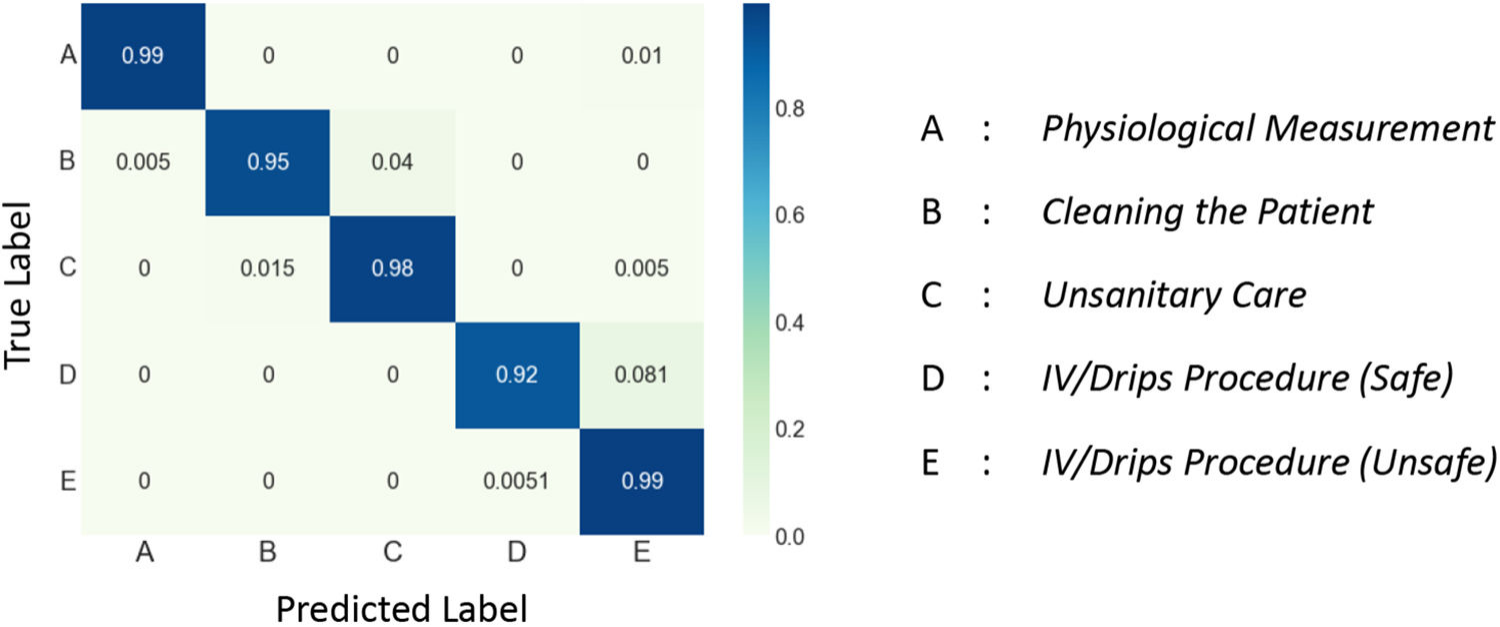
Confusion Matrix of *X-CHAR* on Nurse Dataset.

**Fig. 6.**
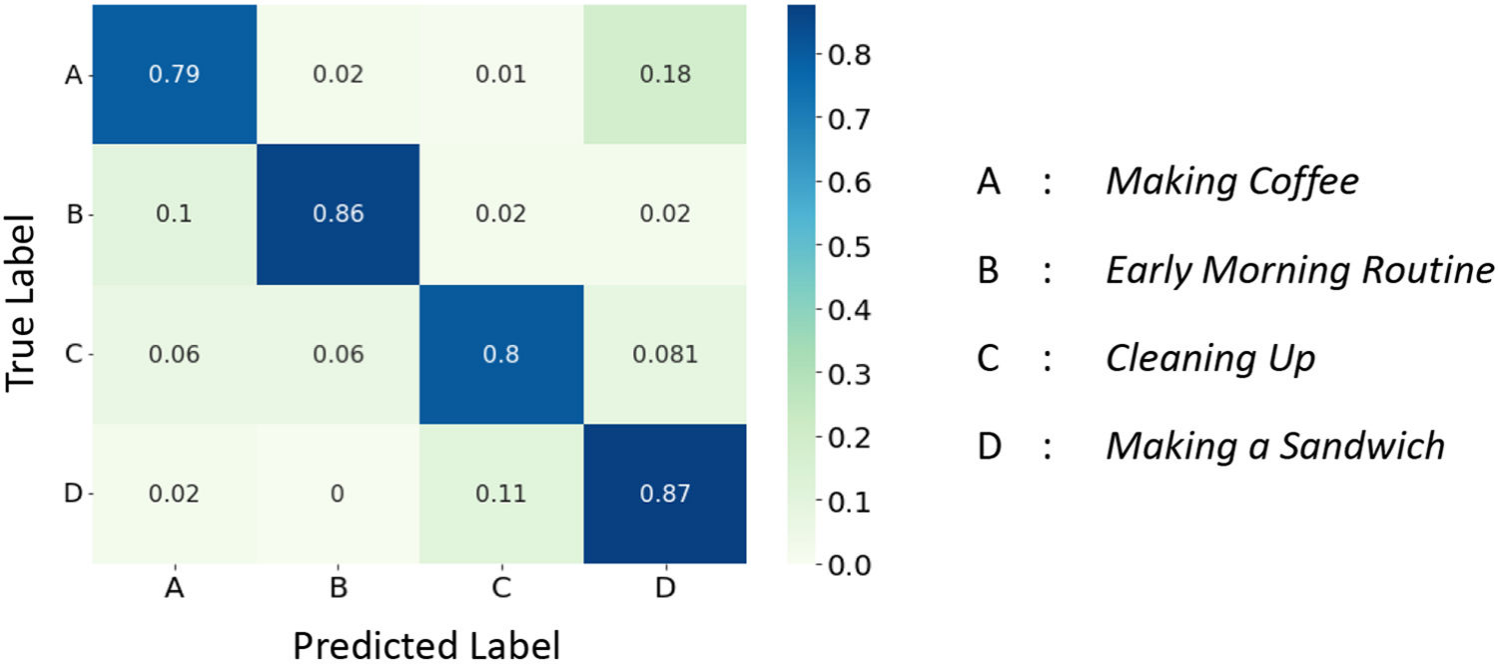
Confusion Matrix of *X-CHAR* on Opportunity Dataset.

**Fig. 7.**
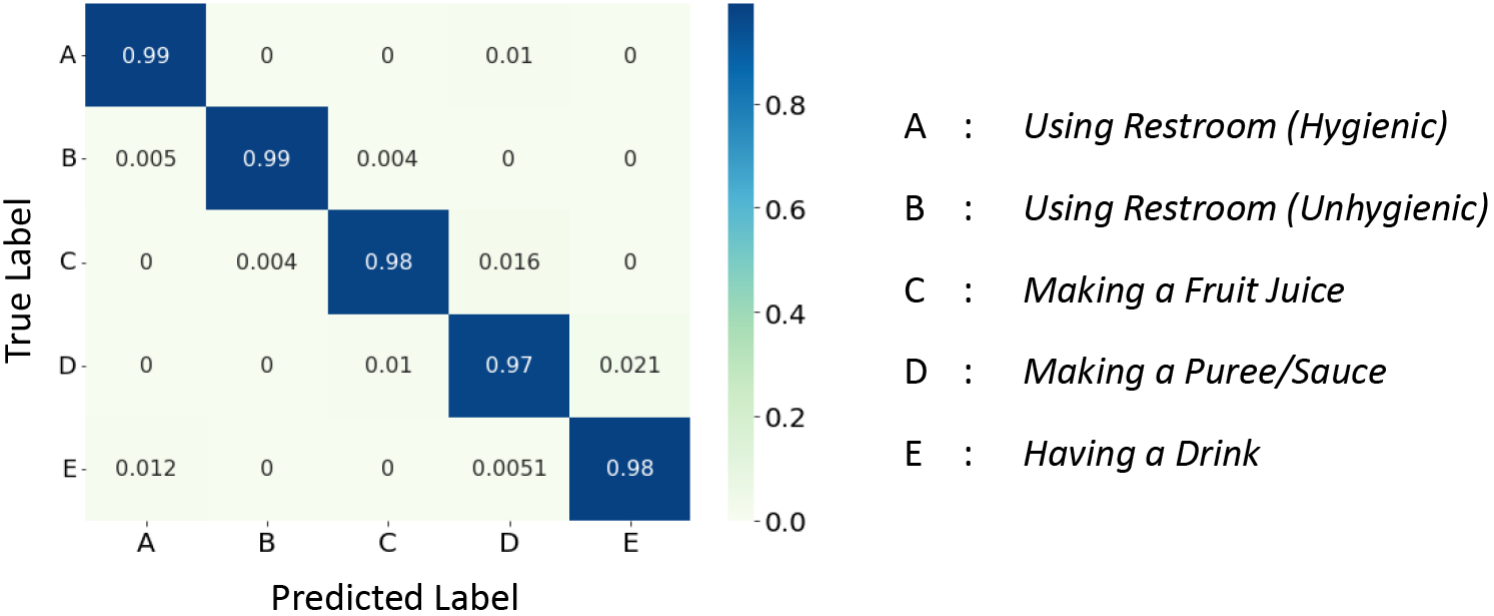
Confusion Matrix of *X-CHAR* on CRAA Dataset.

We first evaluate the performance of *X-CHAR* in complex activity detection on the three datasets. *X-CHAR* achieved a mean F-1 score of 0.97, 0.836, and 0.988 on Nurse Activities, Opportunity, and CRAA datasets, respectively. We observe from the normalized confusion matrix on Nurse and CRAA datasets shown in Figure 5 and Figure 7 that *X-CHAR* achieved a near-perfect prediction performance across all the classes. The normalized confusion matrix for the Opportunity dataset shown in Figure 6 conveys that *X-CHAR* makes some errors in recognizing the ’making coffee’ activity while the rest of the complex activities are predicted with good accuracy.

### 6.2 Comparison of Complex Activity and Concepts Recognition Performance with Baselines

We compared the performance of *X-CHAR* with other complex activity recognition models discussed in Section 5.4 and *X-CHAR* without the temporal bottleneck module on the three datasets. Table 5 shows the task (CHAR) F1-score, concept accuracy, and concept edit distance of all six comparison methods on the three different datasets. We observe that, despite having the temporal bottleneck constraint, *X-CHAR* achieves similar or better task performance to the black-box models that don’t have any bottlenecks (DEBONAIR and X-CHAR without bottleneck) on all three datasets.

*First*, we can see that *X-CHAR* achieves the highest F1-score among all comparison methods for *Nurse Activities* and CRAA datasets, which are 0.9698 and 0.9886, respectively. The F1-score of *X-CHAR* is almost equal to the F1-score of models without bottleneck for the opportunity dataset. Overall it shows that the F1-score of *X-CHAR* is better or similar to all other comparison methods.

*Second, X-CHAR* achieves the best concept prediction accuracy among all the baseline models on all three datasets (89%, 67%, and 97%, respectively). DEBONAIR and X-CHAR without bottleneck models do not predict concepts; hence, they have no concept accuracy reported. CBM cannot capture the multiple occurrences of a concept, and therefore its accuracy is lower than *X-CHAR* . On the other hand, AROMA, as discussed in 5.4, predicts a concept for every small segment independently, which results in it predicting lots of incorrect concepts for a given input. Hence its concept prediction accuracy is poor compared to *X-CHAR* and CBM. The non-end-to-end model (ConvLSTM +TCN) has a decent concept prediction accuracy but a poor complex activity prediction performance. This is a result of training the concept and complex activity models independently.

*Third*, among the baselines, *X-CHAR* , AROMA and ConvLSTM+TCN predict a sequence of concepts. We observe that *X-CHAR* has a much lower mean edit distance when compared to the other two. AROMA’s poor concept performance is because it predicts a concept for each segment independently, which results in incorrect concept predictions, especially in segments that can have multiple concepts (i.e., transitions from one concept to another) or when there is a random motion while performing the complex activity. Therefore concept sequences provided by *X-CHAR* are more precise and hence offer a better explanation of the complex activity.

In summary, we do not observe a trade-off between task prediction performance and high concept accuracy (i.e.) *X-CHAR* does not compromise the classification accuracy (with respect to accuracy and edit distance) while providing explainability for all three datasets.

### 6.3 Qualitative Analysis of Explanations

Here, we discuss the qualitative analysis of the explanations of *X-CHAR* and the other three baseline explanation methods one by one for an instance of the nurse activity dataset. GradCAM and Exmatchina are post-hoc methods, and they will explain the decisions made by the pretrained black-box DNN model without the temporal bottleneck module for complex activity recognition. CBM and *X-CHAR* are inherently interpretable DNN models that provide both complex activity classification and corresponding explanations.

#### 6.3.1 *Explanation from* X-CHAR

**Fig. 8.**
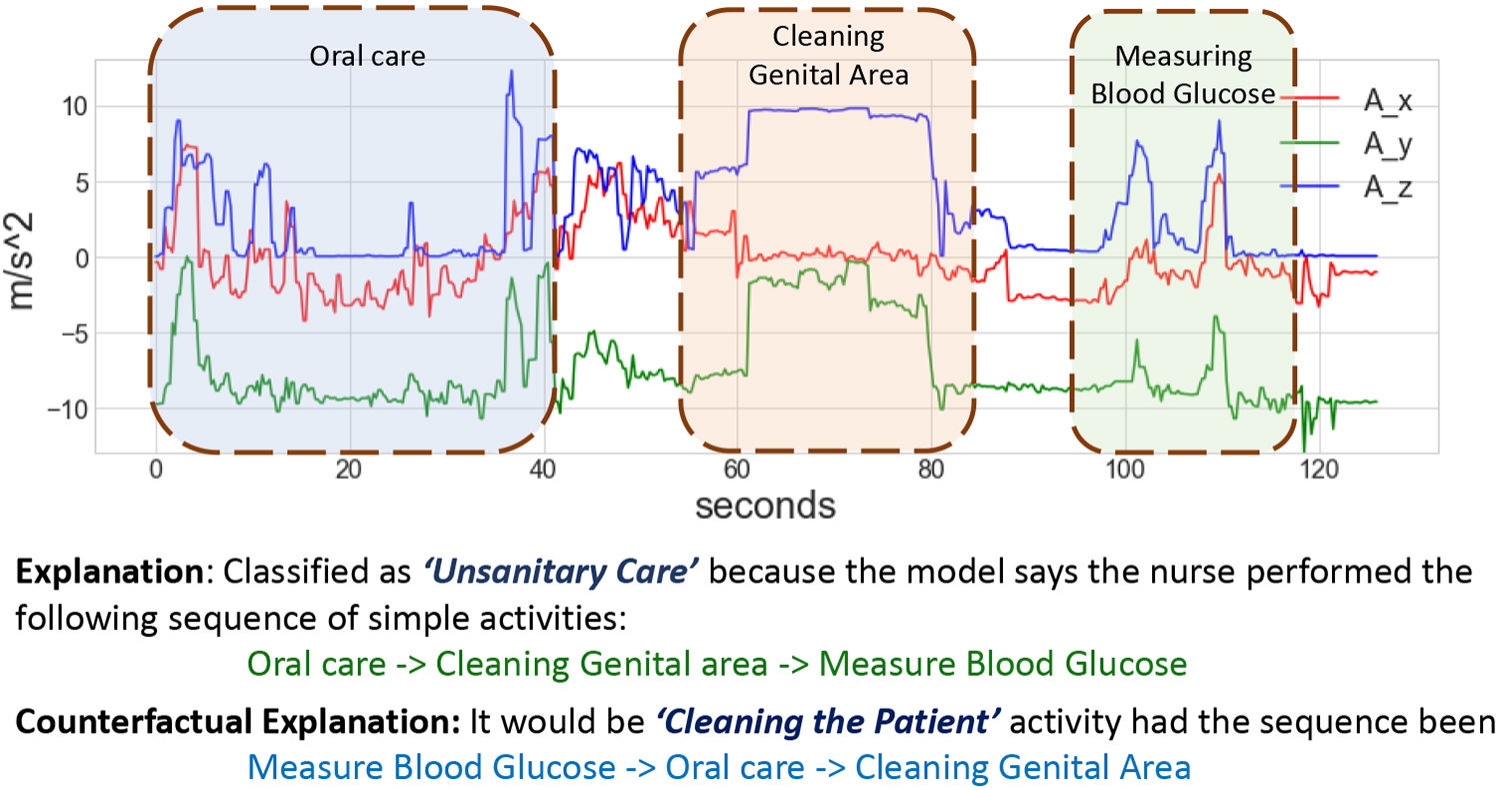
Explanation provided by *X-CHAR* for a test input from Complex Nurse Activities dataset

Figure 8 shows the explanation from *X-CHAR* for a test sample from the nurse activity dataset. Here x-axis and y-axis represent the time (in seconds) and acceleration (in m/s^−2^), respectively. Three lines show x,y, and z-axis values from the accelerator sensor from the smartwatch. As the figure shows, *X-CHAR* first detects three concepts (i.e., oral care, cleaning genital area, and measuring blood glucose) from the raw input signals, as shown using the dashed rectangles. Then, *X-CHAR* classifies the given input instance as *unsanitary care* due to the fact that the nurse performs the *measuring blood glucose* activity before the *cleaning genital area* activity. In addition to the high performance in complex activity recognition, the temporal bottleneck module improves the explainability of *X-CHAR* by providing the concepts and the sequence in which they occur for the given test sample. From the figure, we can also say that *X-CHAR* provides the time duration information for the predicted concepts.

#### 6.3.2 Explanation from GradCAM

**Fig. 9.**
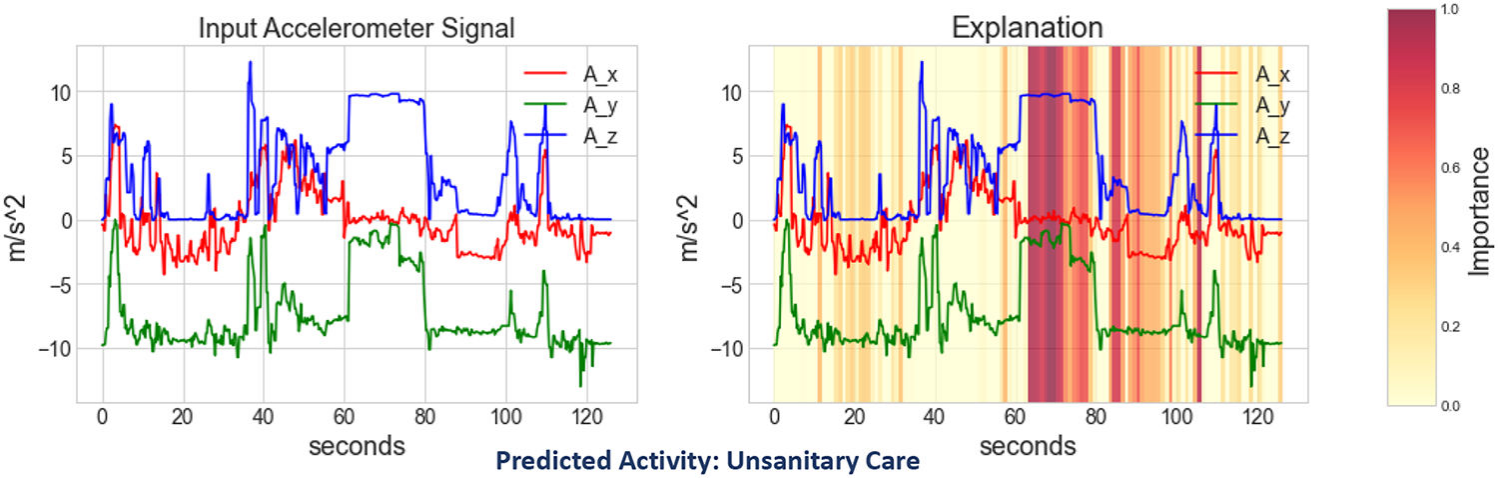
Explanation provided by GradCAM on a trained black-box model for a test input from Complex Nurse Activities dataset.

Figure 9 shows the explanation from GradCAM to test the same sample from the nurse activity dataset (as used in Figure 8). As in the previous figure, the x-axis and y-axis represent the time (in seconds) and acceleration (in m/s^−2^), respectively. Here, the left figure shows the original input, and the right figure shows a heatmap or saliency map highlighting the important region on the input sample for the complex activity classification. More specifically, the right figure shows the significance of different input segments to classify the given input as *unsanitary care* activity by the black-box model (We considered X-CHAR without the temporal bottleneck as the black-box model). However, unlike concept-based explanations, the explanations offered by GradCAM do not provide human-understandable reasoning behind the classified outcome to a non-technical end-user as it only highlights certain regions in the input.

#### 6.3.3 Explanation from ExMatchina

**Fig. 10.**
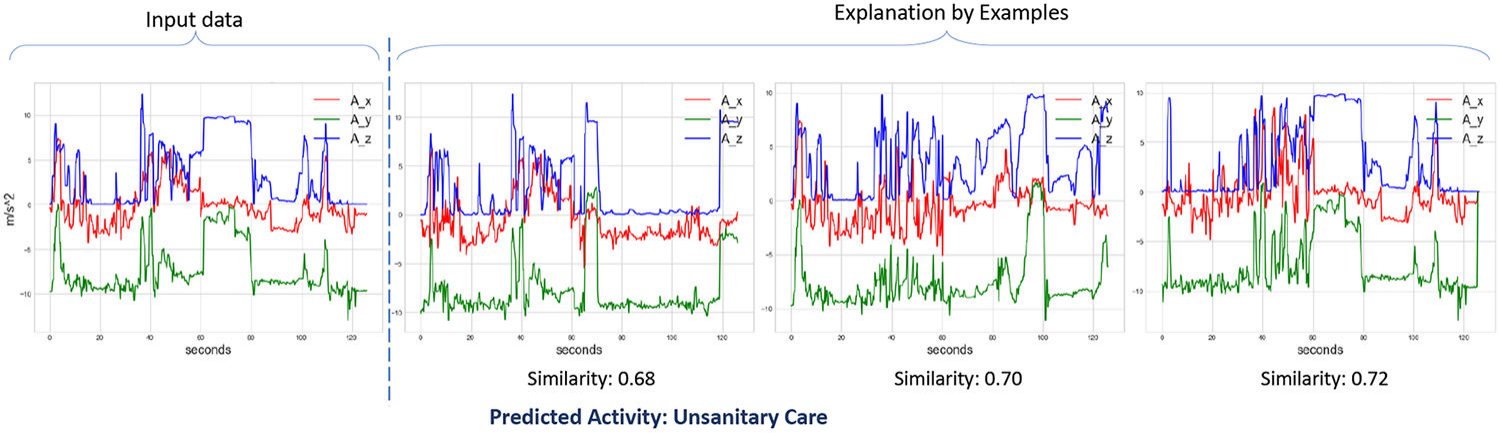
Explanation provided by ExMatchina on a trained black-box model for a test input from Complex Nurse Activities dataset.

Figure 10 shows the explanation from ExMatchina for the same sample from the nurse activity dataset. As before, the x-axis and y-axis represent the time (in seconds) and acceleration (in m/s^−2^), respectively. Exmatchina is an explanation-by-example-based method and shows ’k’ most similar samples from the training dataset that were also classified as the same class as the test input. In Figure 10, the input test sample to the model is shown on the left side of the plot and is classified as *unsanitary care* by the black-box model (We considered X-CHAR without the temporal bottleneck as the black-box model). The explanation, which is the three most similar examples, is shown on the right of the dashed line. From the figure, we observe that the explanations from ExMatchina expect the users to recognize the common patterns between the input and the explanation samples. Though this approach might be helpful for tasks on simple datasets, as shown in the study [29] for complex activity recognition tasks that involve non-readable motion sensor data from multiple sensory channels, this method of explaining by expecting users to pattern match seems counter-intuitive.

#### 6.3.4 Explanation from CBM

**Fig. 11.**
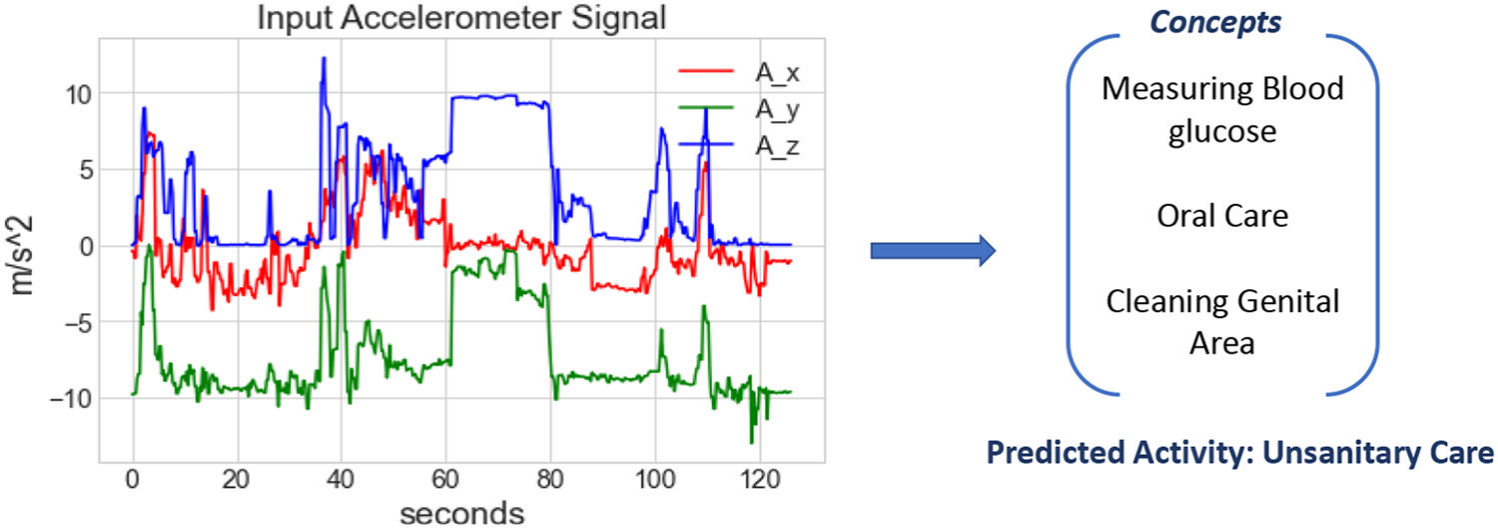
Explanation provided by Concept Bottleneck Model for a test input from Complex Nurse Activities dataset.

Figure 11 shows the explanation from CBM model to test the performance of CBM on the same sample from the nurse activity dataset (as used in previous figures). Similarly, the x-axis and y-axis represent the time (in seconds) and acceleration (in m/s^−2^), respectively. As we see in the figure, CBM offers an explanation in the form of concepts present in that complex activity time duration. Still, it does not provide any information on the sequence or the time in which those concepts occurred. But for complex activity recognition tasks, different complex activities can have the same concepts but a different sequence or the order in which the concepts occur. Therefore, the explanations offered by CBM are not complete as it doesn’t have sufficient information (like the order or time of the concepts) that can fully explain the complex activity.

#### 6.3.5 Explanation from AROMA

**Fig. 12.**
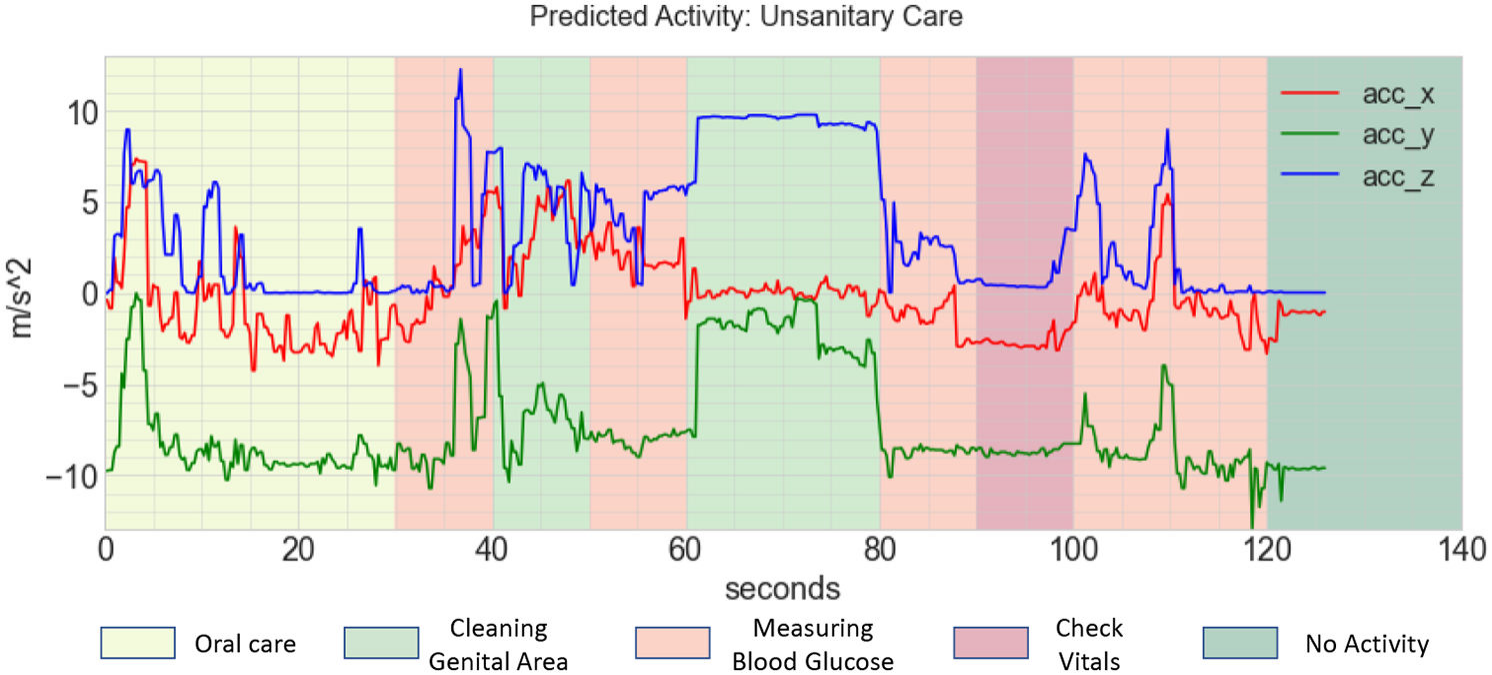
Classification and Explanation provided by AROMA for a test input from Complex Nurse Activities dataset.

Figure 12 shows the explanation from the AROMA model on the same sample from the nurse activity dataset (as used in previous figures). AROMA predicts a concept for every 10-second segment and a complex activity for the given input. In our study, we consider the sequence of concepts provided by AROMA as the explanation for the complex activity. The concepts are represented by different background colors, as shown in the figure. The x-axis and y-axis represent the time (in seconds) and acceleration (in m/s^−2^), respectively. From the figure, we observe that the explanation offered by AROMA is not robust. It mainly suffers because AROMA predicts incorrect concepts in segments where there is a transition of concepts or when there is no activity for most of the segment. This results in a noisy explanation and is not easily understandable to the end user.

#### 6.3.6 Explanation from DeXAR

**Fig. 13.**
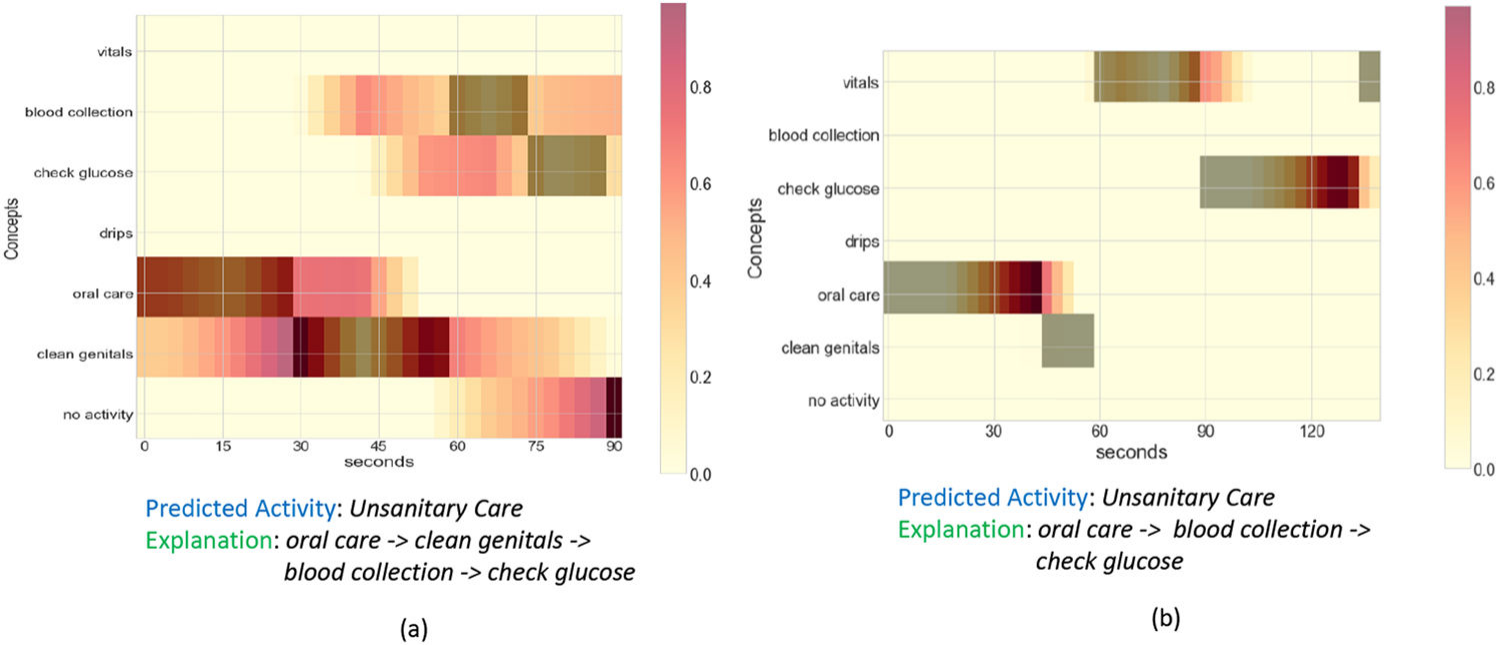
Explanation provided by DeXAR for two test inputs from Complex Nurse Activities dataset.

Figure 13 shows the explanation provided by the DeXAR method on two different samples from the nurse activity dataset. This method converts the sequence of concepts into semantic images, and a CNN classifier is trained on these images to predict the complex activity. Then using post-hoc methods, DeXAR obtains the heatmap of the important concepts responsible for the complex activity classification, as shown in the figure. It also provides a text-based explanation mentioning concepts with an importance score greater than a specific threshold t. However, there are some key limitations that we observed in DeXAR. The threshold ’t’ is chosen empirically; hence, there are cases where the important concepts are missed as they fall below the threshold. In Figure 13, for both samples (a) and (b), we used the same threshold and normalized the importance scores. In (a), we find that the important regions are wider and overlap in the time-axis. This indicates that the order and absence of concepts also matter for the complex activity classification but is not captured in the text-based explanation. In (b), we find that one of the key concepts is not considered important as it falls below the threshold. These limitations make the explanations from DeXAR challenging to comprehend for the end user.

### 6.4 Comprehensibility of *X-CHAR* Explanations

#### 6.4.1 Study Methodology

We conducted three separate Amazon Turk studies, one for each dataset. The goal of the study was to determine which explanation method was the most understandable and preferred by an average end-user for complex activity recognition tasks. Each study was divided into two parts. In the first part, we compared X-CHAR with the methods that provide explanations in the raw input space. In the second part, we compared X-CHAR with explanation methods that operate in the concepts space, as discussed in the previous section. The study survey questions were formed in the following manner: First, the participants were provided information on what complex activity recognition task is being done by the machine learning system. Then, a random test input was selected for each survey question, and the model-predicted class was presented along with explanations from two randomly selected explanation methods. Participants were asked to select which of the two provided explanations was easy to understand and offered a better explanation for the corresponding model’s prediction. Each participant answered a survey containing 12 questions. Since we had 75 participants in total, we had 900 validated responses comparing the explanations for each dataset.

##### Participant Information:

There were 75 participants in our Mechanical Turk studies. Among them, 40 participants did not share the optional demographic information. From those who provided the demographic information, there were 22 participants in the age group *20-30*, 9 participants in the age group *30-40*, and 4 participants in the age group > *40*. We also requested their education levels: no college degree, undergraduate, or graduate degree. 28 participants had an undergraduate degree, 3 had a graduate degree, and four were without a college degree.

#### 6.4.2 Study Results

**Fig. 14.**
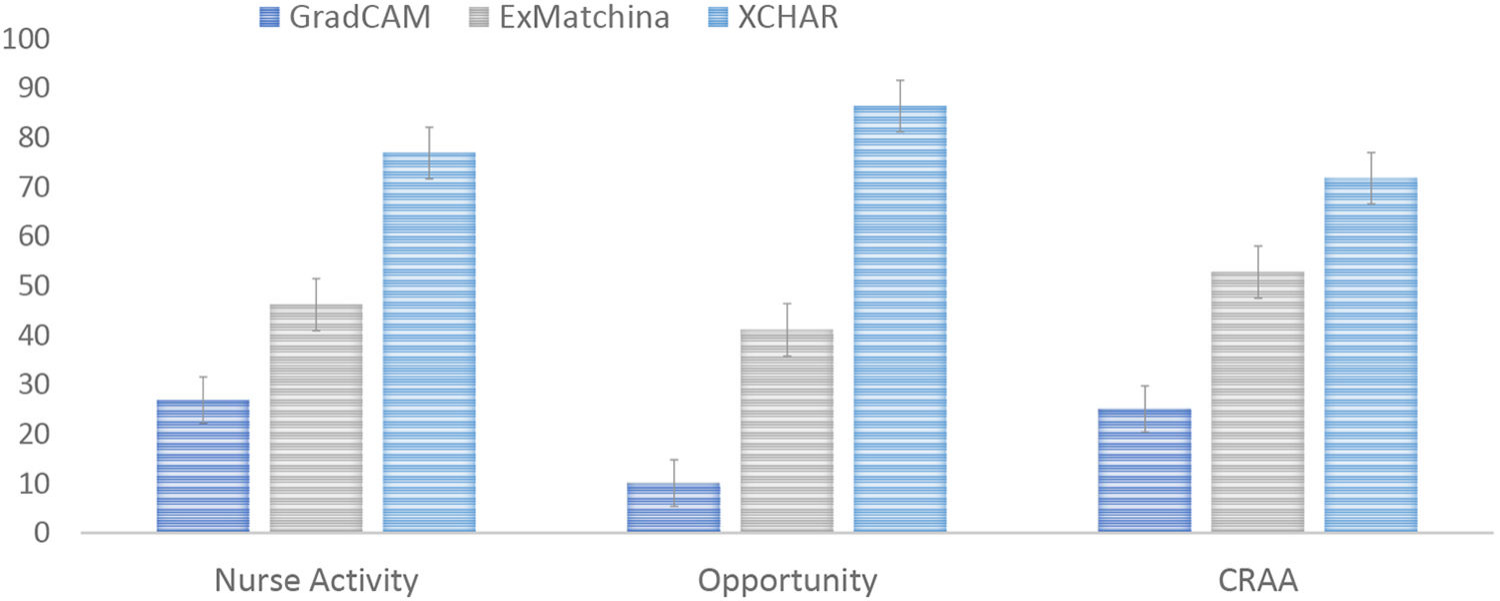
The preferred explainabilities of different models (i.e., GradCAM, ExMatchina, and X-CHAR) operating in raw input space from the Turk study.

**Fig. 15.**
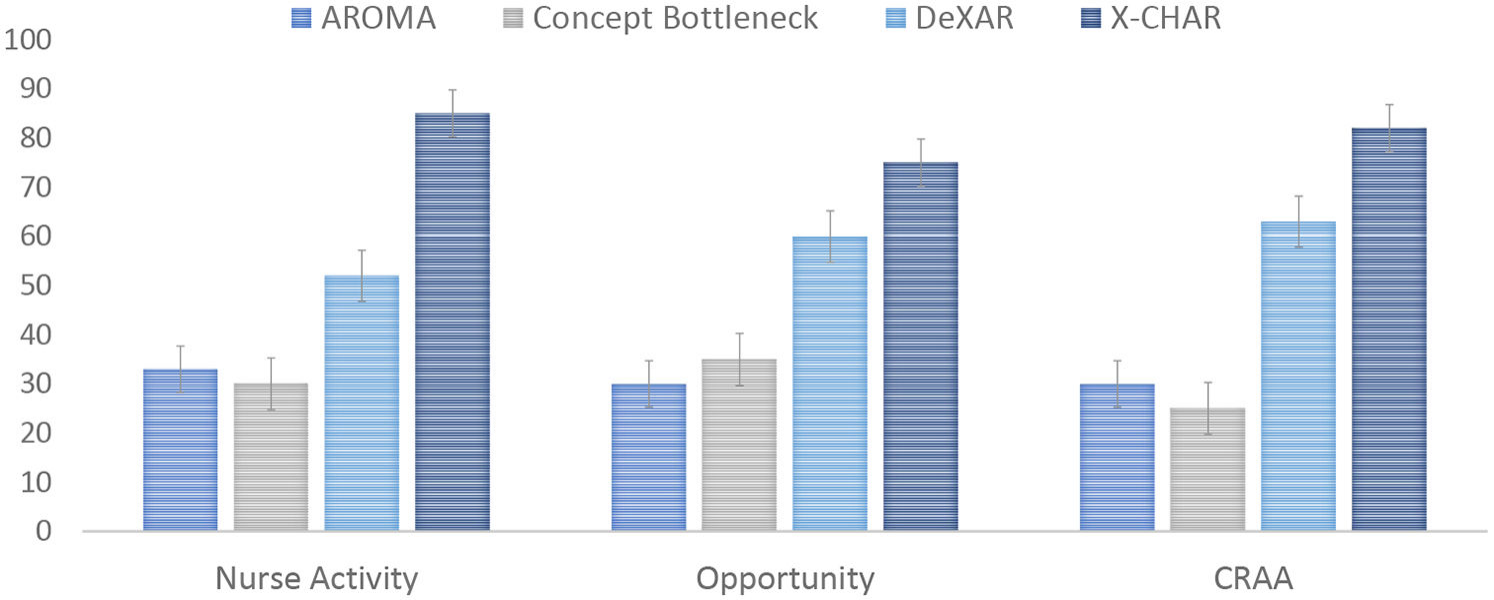
The preferred explainabilities of different models (i.e., AROMA, Concept Bottleneck, DeXAR, and X-CHAR) operating in concept space from the Turk study.

Figure 14 and Figure 15 present the aggregated results of the Mechanical Turk study when comparing *X-CHAR* with (a) explanation methods operating on the raw input space and (b) explanation methods operating on the concept space. The results show that explanations provided by the *X-CHAR* were largely considered as the preferred explanation on all three datasets in both scenarios. In scenario (a), when *X-CHAR* explanation was an available option, the participants selected it 76.9%, 88.4% and 71.8% of the time in Nurse complex activity, Opportunity, and CRAA datasets, respectively. The second preferred method is the explanation by examples, and GradCAM is the least preferred method for complex activity recognition tasks. And in scenario (b), when *X-CHAR* explanation was an available option, the participants selected it 84.8%, 75.6% and 82.5% of the time in Nurse complex activity, Opportunity, and CRAA datasets, respectively. DeXAR was the second preferred method, while explanations from the Concept bottleneck model and AROMA were preferred significantly less. The presented confidence intervals are calculated using the bootstrap method as described in [19] for 95% confidence. Therefore, the results indicate that explanations in the form of concepts are preferred over explanations projected on input space. Also, the explanation from *X-CHAR* is the most preferred method among the other concept-level explanation methods to average end-users who may not possess knowledge of machine learning, i.e., the "non-expert" layperson.

### 6.5 Faithfulness of *X-CHAR* Explanations

As proposed in [17, 26], for an explanation to be faithful, it should satisfy the following property: 1. Two test inputs $x,x^{,}$ that get the same explanation should also have the same prediction, which implies that two test inputs $x,x^{,}$ with different predictions should have different explanations and 2. On similar test inputs where the model made identical predictions, its explanations must be similar.

To verify the first property, we analyzed the explanations and predictions produced by *X-CHAR* on our test dataset and found that no two test inputs had the same explanations with different predictions. In addition, we created a perturbed dataset where each sample in the test dataset was split into smaller segments and shuffled. Then, we passed this perturbed dataset through *X-CHAR* and noticed that the predictions and explanations of the perturbed dataset were different when compared to the predictions and explanations of the original test dataset.

To verify the second property, we added a small amount of gaussian noise (5%) to the test samples and compared their predictions and explanations with the unperturbed test samples. We observed that the explanations from *X-CHAR* remained the same for samples where the predictions remained the same after adding noise. However, for certain samples, the predicted complex activity did change and their corresponding explanations also changed compared to the explanations from the unperturbed test input.

## 7 DISCUSSION

In this section, we first discuss our key observations from our experiments regarding complex human activity recognition tasks. Then, we discuss the current limitations of *X-CHAR* and provide some interesting future research directions to expand on our work.

### 7.1 Key Observations

- **The Myth of Accuracy vs. Interpretability Trade-off.** A common assumption in the literature is that the performance of the machine learning models drops when the models become explainable [1]. This is because they usually consider traditional methods, such as decision trees or linear models while making those comparisons. However, it is not necessarily true. Our work shows that the model developers can design complex deep learning architectures but constrain the latent space to capture human-understandable concepts relevant to the particular task. This way, we can imbue interpretability directly into the models without losing accuracy.
- **Human Preferred Explanations for Sensory Data.** It is already well-known that Sensory data like motion sensors, unlike images or text, is inherently difficult to understand for humans. Also, the data becomes more complex as sensors and sensory channels increase. Only users who are domain experts in the field of that sensor modality can comprehend the values, and it is difficult for a layperson. So the explanation methods that highlight specific portions of the input space expect the users to have the domain knowledge to understand the sensor readings. They tell where the model is looking but leave out all information about how relevant information is being used. Therefore, they are not preferred when explaining to a non-technical end-user. Our results have confirmed the same and have shown that humans prefer explanations in the form of concepts, especially when the input data is not intuitive. We also observed that more study participants preferred concept-based explanations over explanations on input space when the number of sensory channels increased.
- **The Easiness of the Sensory Data Annotation Process.** One of the main drawbacks in collecting data for complex activities is that it is difficult to accurately label the start and end times of the simple activities (concepts) that occur while performing the complex activity. Since *X-CHAR* learns the timing information of the concepts automatically using CTC Loss, we only have to label the sequence of concepts that occurred while performing the complex activity. Therefore, it helps us to discard the laborious efforts of labeling each time step manually.

### 7.2 Limitations

Despite our best efforts, there are a few limitations of our current work as follows:

- When the number of concepts is large, there might be a large number of permutations and combinations of these concepts corresponding to the complex activities. Though, in theory, it is possible to identify all the sequences, in practice, it is difficult to collect data for all the possible permutations.
- In our work, our counterfactual explanations depend on the valid concept sequences from the training dataset. Therefore, we need to store the training data’s concept sequences to generate counterfactuals. This might occupy significant memory for large datasets.
- While *X-CHAR* provides explanations in the form of concept sequences and counterfactual instances, it does not provide us with any importance scores corresponding to the concepts.
- *X-CHAR* mainly applies to interpreting the CHAR at the concept level for a given test input. It does not try to explain the parameters of the DNN-based “black-box" models and hence does not offer information on the internal reasoning of the model.

### 7.3 Future Scope

Based on the above discussions, we list the following future research directions:

- **Concept-based Attention Layer.** Though *X-CHAR* accurately predicts the concepts, it does not particularly highlight the part of the sequence that is important. Therefore, a possible future direction to explore would be to replace the classifier module after the temporal bottleneck module with a concept attention-based layer. This might help the model learn rules from the concepts, resulting in better explanations.
- **Neurosymbolic Architecture.** It is shown that neuro-symbolic programming can be used to identify the valid sequences of concepts (i.e., behaviors) from different environments [10]. Usually, symbolic methods are less complex to aggregate the concepts to realize specific learning tasks. Therefore, a possibility is to replace the final classifier module with a symbolic architecture to search for the desired sequence of concepts when needed. This might also provide some global understanding of the internal workings of the model.
- **Robustness of CHAR Models.** Based on the variable accuracies of our method in different datasets, we assume that there exists a domain shift due to the lack of adequate datasets to cover all possible edge cases. A future research opportunity would be to generate a synthetic activity recognition dataset for all possible scenarios and use it to train a robust CHAR model.

## 8 CONCLUSION

In this paper, we presented *X-CHAR* , an end-to-end, interpretable deep learning model for complex activity recognition. For any given test input, *X-CHAR* provides the sequence of simple activities relevant to the task called concepts and a counterfactual concept sequence as human-understandable explanations. *X-CHAR* consists of three modules: sensor fusion, temporal bottleneck, temporal classifier, and an explanation generator. The temporal bottleneck layer captures the concepts for complex CHAR classification that helps generate explanations in the form of a sequence of concepts alongside counterfactual explanations that are faithful to the model’s decision-making process. As the name suggests, the explanation generator decodes the concept sequence and identifies the counterfactual explanations. We thoroughly evaluated our model on three complex activity datasets and showed that our model achieves state-of-the-art performance (i.e., F1-score and accuracy) in complex activity recognition while providing human-understandable explanations. We also surveyed 75 participants to study the understandability of the explanations offered by *X-CHAR* with respect to the existing methods for complex activity recognition and found that the participants preferred explanations from *X-CHAR* by a statistically significant margin on all three datasets.

## Figures and Tables

**Table 1.** The complex activities, the sequence of concepts corresponding to each complex activity and the list of all concepts present in the Nurse Activities Dataset.

Complex Nurse Activity	Concept Sequence	Concepts
Physiological Measurement	Vitals → Blood collection → Blood Glucose	check vitals, collect blood, check blood glucose, oral care, clean patient, drips
	Blood collection → Vitals → Blood glucose	
Patient cleaning	Vitals → Oral care → Clean	
	Oral care → Vitals → Clean	
	Oral care → Blood glucose → Clean	
	Blood glucose → Oral care → Clean	
Unsanitary Operations	Clean → Blood glucose → Vitals	
	Clean → Oral care → Vitals	
	Vitals → Clean → Oral care	
	Oral care → Clean → Blood glucose	
Safe IV/Drips Procedure	Vitals → Blood collection → Drips → Vitals	
	Vitals → Drips → Vitals	
	Vitals → Drips → Blood collection → Vitals	
Unsafe IV/Drips Procedure	Vitals → Blood collection → Drips	
	Blood collection → Drips → Vitals	
	Drips → Blood collection → Vitals	

**Table 2.** The complex activities and concepts for the Opportunity dataset.

Complex Activities	Concepts
Making coffee, Early morning routine, Cleaning up, Making a sandwich	move item, reach item, release item, use item, open door, close door, open fridge, close fridge, open dishwasher, close dishwasher, open drawer, close drawer, switch on, switch off, drink, bite, clean

**Table 3.** The complex activities, the sequence of concepts corresponding to each complex activity and the list of all concepts present in the CRAA: Complex Restaurant Activities from Audio dataset.

Restaurant Activity	Concept Sequence	Concepts
Using Restroom (Hygienic)	Footsteps → Using toilet → Toilet flush → Wash Hands	footsteps, using toilet, flush toilet, wash hands, open shelf, chopping vegetables, peeling, using blender, take glass, pour water, drinking
	Wash Hands → Using toilet → Toilet flush → Wash Hands	
Using Restroom (Unhygienic)	Footsteps → Using toilet → Toilet flush	
	Footsteps → Wash Hands → Using toilet → Toilet flush	
	Wash Hands → Using toilet → Toilet flush → Footsteps	
Making a fruit juice	Opening shelf → Chopping → Using blender	
	Peeling → Chopping → Using blender	
	Opening shelf → Peeling → Using blender	
Making a puree/sauce	Peeling → Using blender → Chopping → Using blender	
	Chopping → Using blender → Peeling → Using blender	
	Open Shelf → Using blender → Chopping → Using blender	
Having a drink	Take glass → Pour water → Drink	

**Table 4.** The *X-CHAR* architecture.

Module	Layers
Sensor Fusion	No. of Sensors x 1-D Convolutional layers Filters: 64, Kernel size: 16, Stride: 2, Activation: ReLu
	1-D Convolutional layer Filters: 128, Kernel size: 16, Stride: 2, Activation: ReLu
Temporal Bottleneck	Bi-Directional LSTM layer LSTM units: 128
	Time Distributed Dense Layer Neurons: No. of concepts, Activation: Softmax
Classifier	Temporal Convolution Layer Filters: 64, Kernel size:8, Stride:1, Activation: ReLu
	Dense Layer Neurons: No. of Classes, Activation: Softmax

**Table 5.** Performance comparison of X-HAR with other baseline models on the three datasets.

Dataset	Model	Task F1-score	Concept	
			Accuracy	Edit Distance
Nurse Activities	DEBONAIR	0.9681	-	-
	ConvLSTM + TCN	0.9274	76.38	2.01
	AROMA	0.9477	72.25	2.26
	CBM	0.9244	87.40	-
	X-CHAR	0.9698	**89.35**	**0.15**
	X-CHAR (no-bottleneck)	0.9695	-	-
Opportunity	DEBONAIR	0.8362	-	-
	ConvLSTM + TCN	0.7956	60.24	1.98
	AROMA	0.8218	53.97	2.87
	CBM	0.7726	65.52	-
	X-CHAR	0.8357	**67.36**	**1.54**
	X-CHAR (no-bottleneck)	0.8382	-	-
CRAA	DEBONAIR	0.9880	-	-
	ConvLSTM + TCN	0.9272	90.08	1.20
	AROMA	0.9576	86.40	1.94
	CBM	0.9032	94.58	-
	X-CHAR	0.9886	**97.70**	**0.10**
	X-CHAR (no-bottleneck)	0.9886	-	-

## Box

CCS Concepts: **• Human-centered computing → Ubiquitous and mobile computing systems and tools; • Computing methodologies → Supervised learning; Neural networks**.
